# Use of OCT Angiography in Choroidal Melanocytic Tumors

**DOI:** 10.1155/2017/1573154

**Published:** 2017-10-19

**Authors:** Juan J. Toledo, Mónica Asencio-Duran, Jesús R. García-Martinez, Alejandro López-Gaona

**Affiliations:** ^1^Ophthalmology Department, Hospital Universitario Nuevo León, Monterrey, NL, Mexico; ^2^Ophthalmology Department, Hospital La Paz, Madrid, Spain

## Abstract

**Objective:**

To describe OCTA findings in choroidal melanocytic tumors, especially the microcirculation patterns, and to try to correlate with the histopathological studies.

**Methods:**

Cross-sectional, comparative, observational study. 70 cases, including 55 choroidal nevi and 15 choroidal melanomas. Three different observers evaluated specific variables in the choriocapillaris layer on AOCT images and searched for images which described histopathologic vascular patterns, and also, a general description of the images was made. Complementary multi-imaging studies included EDI SD-OCT, color and autofluorescence fundus imaging, Doppler ultrasound, and indocyanine/fluorescein angiography.

**Main Results:**

Good quality studies were acquired in 80% of the cases, with kappa indexes 0.768–0.958. Nevus OCTA images were described mainly as hyperreflective (72.7%), whereas choroidal melanoma as iso/hyporeflective (62.5%). Avascular areas were found in 50.96% and in 33.3% of choroidal nevus and choroidal melanomas, respectively. A neovascular membrane was found only in cases of choroidal nevus (16.3%). Only in cases of choroidal melanomas, we found vascular loops (6.6%) or vascular networks (6.6%).

**Conclusion:**

OCTA is a promising new technology that can be used to study in vivo the differential characteristics of microcirculations between posterior segment melanocytic lesions. Today, larger studies are needed to corroborate these findings and to correlate it with malignancy.

## 1. Introduction

In the past years, with the new Fourier domain OCT equipment including features such as enhanced depth imaging (EDI), there has been a great advance in the diagnosis and follow-up of melanocytic tumors [1–3].

OCT angiography (OCTA) is a new technology that creates flow/structure images, analyzing differences in intensity and phase information of repeated B-scans [4, 5]. Maloca et al. made a pilot study and found that studying melanocytic lesion with OCTA is possible [6].

Choroidal nevi are benign lesions, and their incidence varies between 1.4% in Asians and 6.5% in Caucasians according to several studies [7, 8]. Clinically, the main concern about nevi is to distinguish them from uveal melanoma. A typical choroidal nevus is asymptomatic and is found in a routine dilated eye exam with ophthalmoscopy. These lesions require no treatment but may eventually turn into a melanoma, so it is advisable to periodically monitor them.

Uveal melanoma is the most common malignant intraocular tumor in adults, which accounts for 3.7% of all melanoma cases. US incidence is 6 cases per million and is slightly more common in men with a male/female ratio of 1.29 [9]. Since the fourth Collaborative Ocular Melanoma Study (COMS) report was published in 1997, accurate diagnosis and early treatment of choroidal melanoma became more important due to its known capacity to spread micrometastasis in an early stage of the disease [10]; some published papers state that the smaller the lesion is when diagnosed and treated, the better the long-term life prognosis could be [11]. It is difficult to diagnose melanomas in an early stage because these lesions may seem to be atypical nevi when they are small, and most ocular oncologists may not treat them promptly because of irreversible side effects on visual acuity produced by treatments, especially if the lesion is near the optic nerve or macula. Another concern regards malignancy confirmation, which can be difficult in choroidal melanoma because biopsy sampling techniques are invasive and have several risks; also, inadequate sampling and cytopathologic interpretation may be a major concern [12], so currently, most of the melanocytic lesions are treated based on clinical criteria [13, 14].

*Circulation of Melanocytic Lesions*. The microcirculation of benign nevi is very similar to the surrounding choroidal plexus and is defined as “normal vessels” [15], but they can also show avascular areas (26.1%), straight vessels (39.1%), and parallel vessels (17.4%) [16]; in contrast, uveal melanomas have more complex vascular patterns, like straight vessels with anastomosis, vascular loops, plexus, and vascular arcs [15, 16] in addition to the vascular patterns found in choroidal nevi. Also, some melanoma's microvascular patterns such as vascular loops and networks are more related to metastasis [17–19]. All this could be an opportunity to study the circulation of these tumors with OCTA to improve the diagnosis process and treat them in earlier stages.

Vascular patterns of melanocytic lesions have been studied in a clinical trial with confocal indocyanine green angiography [20], but there are no studies using OCTA.

Our study's main purpose is to report OCTA findings in melanocytic tumors and correlate these findings with the information previously reported by histopathological studies.

## 2. Material and Methods

A total of 68 patients with melanocytic lesions were included; we performed OCTA using Cirrus 5000 OCT (Angioplex by Zeiss); Cirrus 5000 uses 68000 scans per second and is centered at a wavelength of 840 nm. OCTA images are acquired with a complex algorithm that analyzes differences in intensity and phase information called OCT microangiography complex (OMAG) [5].

### 2.1. Image Acquiring Method

We included patients who had tumoral pathology in the posterior pole of the ocular globe, which are suitable for imaging with OCT equipment. We captured several OCTA 6 × 6 mm cubes, including all the lesion when possible. If the lesion was too big to fit in one 6 × 6 mm image, we captured several images and then made a composed picture of the lesion; surrounding “healthy” tissue was always included.

We included 68 cases including 55 choroidal nevi and 13 choroidal melanomas.

Images were evaluated by three different observers who searched for images which described histopathologic vascular patterns, and also, a general description of the images was made.

This study was accepted by La Paz University Hospital's review board, under the code HULP PI-2158 of 10/2015. None of the authors has any conflicts of interest to disclose.

## 3. Results

**Figure 1:**
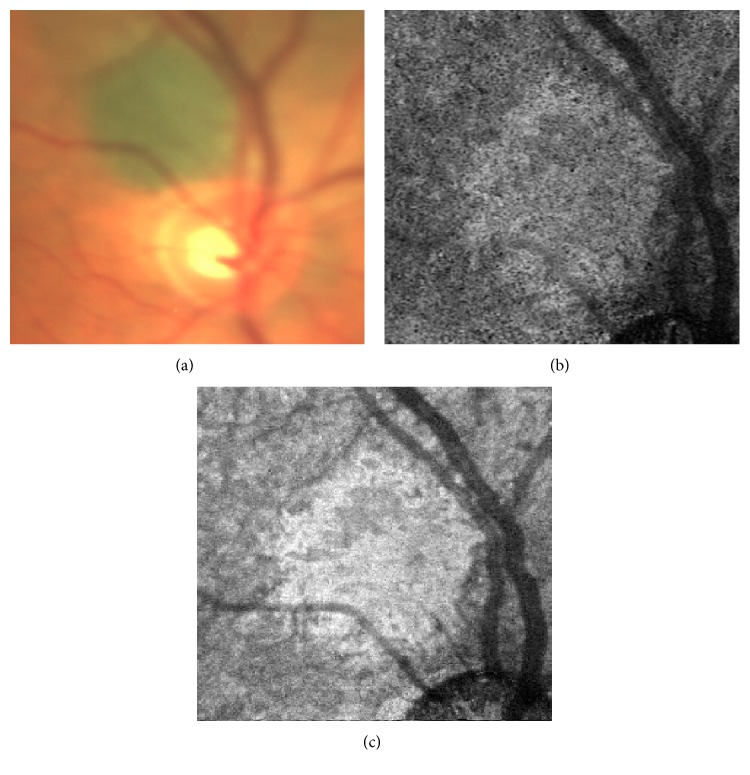
(a) Color fundus photography shows a juxtapapillary choroidal nevus. (b) OCTA choriocapillaris 3 × 3 mm layer shows a hyperreflective homogeneous plexus and well-defined borders. (c) En face OCTA choriocapillaris 3 × 3 mm layer shows a hyperreflective image over the lesion.

**Figure 2:**
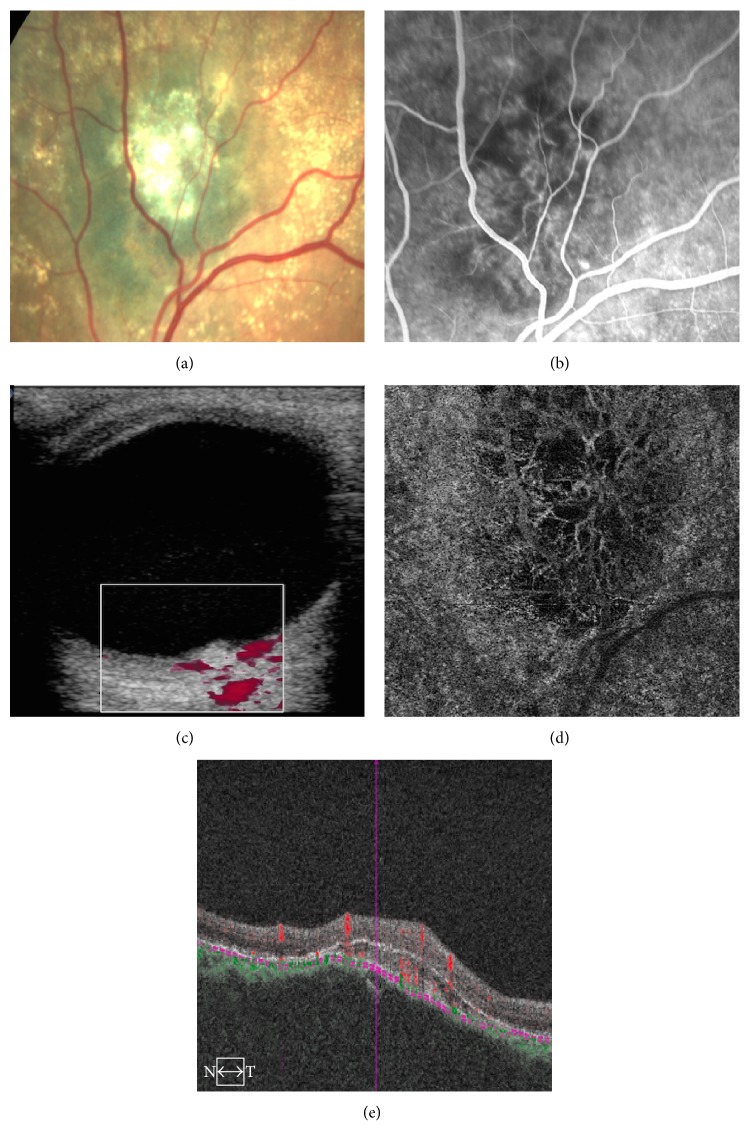
(a) Fundus color photography shows suspicious melanocytic lesion. (b) Fluorescein angiography shows vessels in the area corresponding the melanocytic lesion simulating a “double circulation.” (c) Doppler echography shows high flux “in the inner portion of the lesion.” (d) OCTA “choriocapillaris” 6 × 6 mm layer shows a vascular network in the area corresponding the lesion, but when we check the OCTA B map (e), it shows that vessels are over the lesion corresponding to a neovascular membrane.

We found that technically acquiring OCTA images of choroidal nevi is relatively easy when the lesion is located in the posterior pole or just outside the retinal vascular arcades and has less than 2 mm of thickness and when the patient holds a good visual acuity. We did not find disturbances in retinal OCTA layers over the lesions. In most of our cases, the choriocapillaris layer showed a hyperreflective plexus; we also found that in some cases, avascular areas are associated with neovascular membranes and hyporeflective artifacts due to superficial disturbances (Figures 1 and 2). When we tried to correlate OCTA findings with histopathological studies, we hypothesize that the “normal vessels” described, in OCTA, appear as the thick fine hyperreflective plexus shown in majority of our cases; avascular areas were found in 28 cases (50.9%), neovascular membranes were found in 9 cases (16.3%), and we did not find images that could be correlated with “parallel vessels” or “straight vessels.”

**Figure 3:**
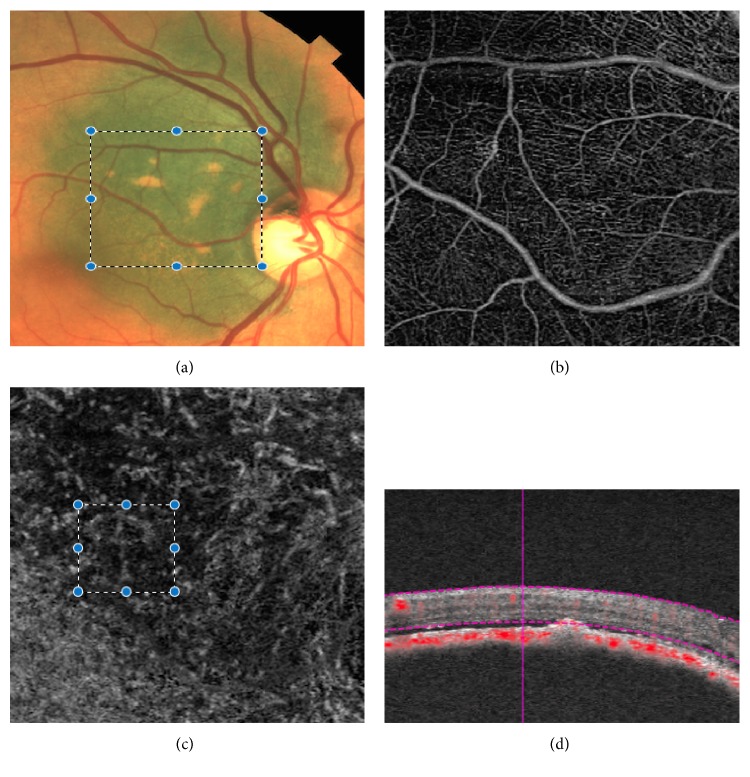
(a) Color fundus photography shows juxtapapillary choroidal melanoma (this tumor has documented progression); marked area corresponds to OCTA images. (b) OCTA superficial 3 × 3 mm layer shows a normal vasculature. (c) OCTA choriocapillaris 3 × 3 mm layer shows a hyporeflective plexus, with thick disorganized vessels. We hypothesize that marked area could correspond to a vascular network formed by several vascular loops. This pattern is associated with aggressive choroidal melanomas. (d) OCTA B map shows a high flux over the inner face of the tumor.

Imaging choroidal melanomas with OCTA is technically difficult; we acquired evaluable images in 8 patients (53.3%). This low index is because of a high number of patients with ocular melanoma who have large masses, retinal detachment, peripheral masses, noncooperative patients, or media opacity. But when we split the group and focused in patients with melanocytic suspicious lesions in the posterior pole with less than 3 mm in thickness, this percentage rises to 87.5%. We found that the vascular plexus of melanomas is much more heterogeneous than nevi's plexus. We found 1 case with images compatible with the “vascular loop” pattern and 1 case with images compatible with the “vascular network” pattern; no images were found suggesting any of the rest of the histopathologic vascular patterns for uveal melanoma (Figure 3). Avascular zones were reported in 5 (33.3%) cases, and no neovascular membranes are reported Table 1.

## 4. Discussion

OCTA seems to be a useful tool to evaluate small chorioretinal tumors; to our knowledge, there are only two studies that evaluate circulation in chorioretinal tumors.

Maloca et al. used Topcon DR1 OCT-1 Atlantis, 1050 nm swept source OCTA, and studied 25 small melanocytic lesions; their goal was limited showing that OCTA technology was able to image and create 3D vascular maps of small melanocytic lesions. In contrast to our study, we used a Zeiss Cirrus 5000 OCT, which is a clinical OCTA equipment with no special software aimed for research.

We found that using OCTA to study choroidal melanocytic tumors is feasible in some cases; it is easier when the studied tumor has a thickness less than 3 mm and is located in the posterior pole. The patient holds a good visual acuity when we have a good patient cooperation. When studying choroidal melanomas, we found that only in 53.3% of the lesions we could obtain a well-performed study, but in melanomas with less than 3 mm thickness, the study could be well performed in 87.5% of the cases. This is important because in this group we included small melanomas which are the hardest to distinguish from atypical choroidal nevi. Mäkitie et al. [21], from the tissue perspective, support the theory that both microvascular densities by counting tumor vessels in a masked fashion from areas of the highest vessel density after immunostaining for CD34 epitope, factor VIII-related antigen (FVIII-RAg), and alpha-smooth muscle actin (SMA) and microvascular patterns contribute independently to prognosis in uveal melanoma in addition to cell type and size of the tumor.

Regarding the study of vascular patterns, we found a hyperreflective plexus in most of the choroidal nevi; avascular areas can be also detected with OCTA. When studying choroidal melanomas, we only found images suggesting vascular patterns in 2 patients. We hypothesize that although OCTA is able to image at least some vascular patterns in choroidal tumors, in our study, it was difficult to obtain images with the sharpness required due to several problems, including the deepness of the tissue analyzed, artifacts that are shallower tissue projects, and the high pigmentation in RPE and in some of the melanocytic tumor. These findings will have to be analyzed in the long term by means of the repetition of new OCTA images in these patients, and the results will be then evaluated in future papers.

## 5. Conclusion

Optical coherence tomography angiography is a new promising technology, as it is an ambulatory, noninvasive, reproducible, quick, and simple exam. Although with current clinical OCTA equipment, it is very difficult to image with full details the circulation of melanocytic lesions. We believe that as this technology advances and improves better, images will be obtained and their use in the daily clinical activities will be possible.

## Figures and Tables

**Table 1:** OCTA findings in melanocytic lesions.

	Number of patients	Choriocapillaris OCTA plexus description	Vascular patterns	Patients where OCTA could be well performed
Choroidal nevus	55 (100%)	Hyporeflective, 2 (3.6%)	Avascular areas, 28 (50.9%)	48 (87.27%)
		Isoreflective, 6 (10.9%)	Neovascular membranes, 9 (16.3%)	
		Hyperreflective, 40 (72.7%)		

Choroidal melanoma	13 (100%)	Hyporeflective, 3 (37.5%)	Vascular loops, 1 (6.6%)	8 (53%)
		Isoreflective, 2 (25%)	Vascular networks, 1 (6.6%)	<3 mm thickness, 8 (87.5%)
		Hyperreflective, 3 (37.5%)	Avascular zones, 5 (33.3%)	
